# Proton transfer induced excited-state aromaticity gain for chromophores with maximal Stokes shifts†

**DOI:** 10.1039/d4sc04692g

**Published:** 2024-10-02

**Authors:** Dong Xing, Florian Glöcklhofer, Felix Plasser

**Affiliations:** a Department of Chemistry, Loughborough University Loughborough LE11 3TU UK f.plasser@lboro.ac.uk +44 (0)1509 226946; b Institute of Applied Synthetic Chemistry, TU Wien Getreidemarkt 9/163 1060 Vienna Austria

## Abstract

Excited state aromaticity (ESA) offers a fascinating route for driving photophysical and photochemical processes but is challenging to harness fully due to its inherent association with unstable antiaromatic ground states. Here, we propose to circumvent this problem *via* the introduction of a new class of photophysical processes, the generation of ESA *via* an excited-state intramolecular proton transfer. We select twelve candidate molecules based on the cyclobutadiene and pentalene scaffolds and investigate their ground and excited state properties using computation. The study highlights the feasibility of proton transfer induced ESA gain and shows that it gives rise to pronounced excited-state relaxation producing Stokes shifts in excess of 2 eV. The underlying electronic structure properties are analysed in terms of the orbitals involved as well as aromaticity descriptors illustrating the pronounced changes these molecules undergo upon both excitation and proton transfer. In summary, we believe that the present work will pave the way toward a new class of chromophores with maximal Stokes shifts and excited-state relaxation.

## Abstract 2

This work introduces a new type of photophysical process, the generation of pronounced excited-state aromaticity *via* a proton transfer. The chromophores developed are distinguished by remarkable Stokes shifts.

## Graphical abstract

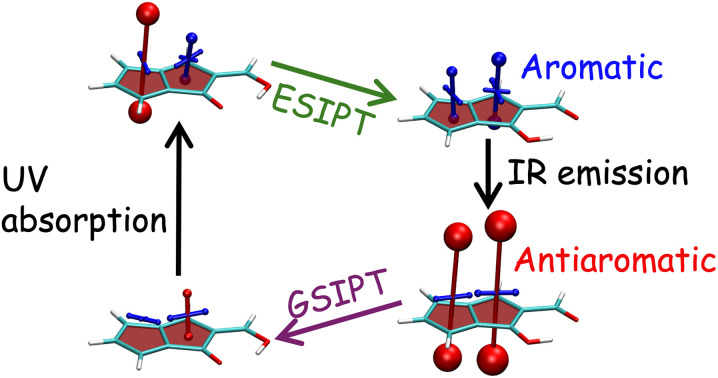

## 1 Introduction

Aromaticity presents itself as an extremely powerful concept in chemistry providing insight into molecular properties far beyond what would be achievable based on simple considerations of valence and connectivity.^1,2^ In its most foundational form, aromaticity is associated with Hückel's rule stating that molecules with [4*n* + 2] π electrons are aromatic and display high stability, whereas those with [4*n*] π electrons are antiaromatic and often highly unstable.^3^ Following Baird's rule,^4^ this relation is inverted in the excited state where molecules with [4*n* + 2] π electrons are found to possess antiaromatic character and excited-state aromaticity is found for [4*n*] π electrons.^5–9^ Within Baird aromaticity, the unpaired spins are delocalised within a cyclic [4*n*] π-system. As an alternative, excited state Hückel aromaticity, where the unpaired spins are outside the π-system, and mixed forms have been reported allowing for the development of a wide range of excited-state aromatic molecules.^10–13^ (Anti)aromaticity is a critical driving force for photochemistry and photodynamics, manifested in the forms of either excited state antiaromaticity (ESAA) relief or excited state aromaticity (ESA) gain.^14–17^ As a paradigmatic example, benzene, possessing excited-state antiaromaticity in its S_1_ and T_1_ states, undergoes rearrangement to relieve the antiaromaticity and isomerize to benzvalene or fulvene.^15,18^ Conversely, ESA gain acts as a driving force for ring planarization in ππ* states.^19^ More generally, considerations of (anti)aromaticity in excited states have been used to design molecular switches,^20–23^ singlet fission dyes,^24–27^ red emitters,^28^ and light-melt adhesives,^29^ highlighting the wider utility of these ideas in driving concrete advances in molecular materials science.

While ESAA relief is a ubiquitous process, which can be expected whenever an aromatic molecule is excited, it is more challenging to design a molecule experiencing pronounced ESA gain considering that this would normally be associated with a highly unstable antiaromatic ground state. However, the development of molecules with pronounced ESA gain is not only fascinating from a fundamental science perspective but it would also provide an important addition to the photophysical toolbox. For example, such molecules could be associated with particularly large Stokes shifts, which are desirable for various luminescence applications^30,31^ as well as for laser dyes.^32,33^ Conversely, if the energetic relaxation is so large that efficient non-radiative deactivation occurs, then this provides a prerequisite for photoprotection or photothermal applications.^34–37^

It is the goal of this work to design molecules with maximal ESA gain after photon absorption. We aim to achieve this target *via* excited-state intramolecular proton transfer (ESIPT). In photochemistry, ESIPT is a seemingly simple but extremely versatile process.^38–41^ Due to their excellent photophysical properties, ESIPT molecules have a range of applications, including in organic light-emitting diodes (OLEDs),^39,42^ molecular sensors,^43–45^ biological imaging agents,^46,47^ molecular logic gates,^48^ and optical memory.^49^ In addition, ESIPT can also be applied in synergy with a variety of other processes, such as aggregation-induced emission (AIE)^50,51^ and thermally activated delayed-fluorescence (TADF).^52^ By combining the ESIPT process with pronounced ESA generation we hope to open new unexplored paths harnessing the desired properties from both.

**Fig. 1:**
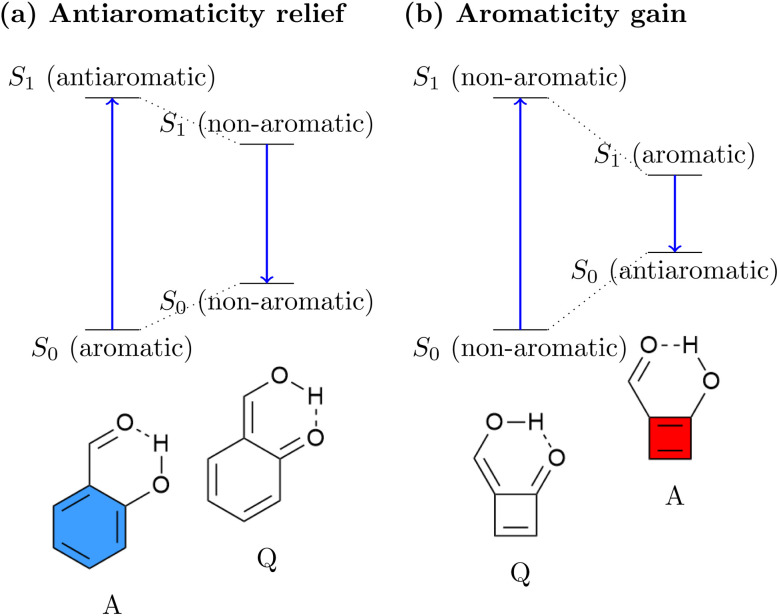
Comparison of (a) standard ESIPT systems driven by antiaromaticity relief and (b) inverted ESIPT systems driven by aromaticity gain as proposed in this work.

The relation between ES(A)A and ESIPT is highlighted in Fig. 1. Generally, an ESIPT molecule includes both proton donating and accepting groups connected through a hydrogen bond. Upon photoabsorption the proton is shifted along the hydrogen bond in an ultrafast dynamical process producing a tautomer of the original molecule. It has been noted that ESAA relief can be seen as the driving force for a range of ESIPT systems.^53–57^ This idea is illustrated in Fig. 1a using the *o*-hydroxybenzaldehyde (OHBA) molecule^58^ as an example. In the ground-state equilibrium structure, OHBA possesses an aromatic ring, shown as a blue sextet, along with a phenolic OH group. We denote this form as the aromatic (A) tautomer. Upon excitation, the lowest singlet ππ* state of the system is accessed. It is antiaromatic *via* Baird's rule providing a strong driving force for ESAA relief. Crucially, the OHBA molecule possesses a unique channel for ESAA relief: it can transfer a proton along its intramolecular hydrogen bond to produce the quinoidal (Q) tautomer. Following this, the conjugation is broken, meaning that the molecule becomes non-aromatic, which is energetically favourable in the excited state. From the non-aromatic excited state of the Q tautomer the molecule can emit a photon reaching the non-aromatic ground state of the Q tautomer. Within the ground state the proton is transferred back to restore aromaticity within the A form, completing the cycle.

While essentially all known ESIPT systems follow the ESAA relief process in Fig. 1a, we want to explore the possibility of inverting the process and to drive ESIPT *via* ESA gain. This process is schematically shown in Fig. 1b. Using a [4*n*] π-electron system, we can expect that its Q tautomer is more stable in the ground state, as the A tautomer would be antiaromatic. Upon excitation, the molecule would experience a significant driving force to transfer a proton, thus generating Baird aromaticity. We believe that this is a fascinating new possibility for a photophysical process with the potential to produce exceedingly large excited-state driving forces and Stokes shifts.

In this work we address the feasibility of designing molecules combining ESA gain with ESIPT following the general scheme of Fig. 1b. We use cyclobutadiene (CBD) and pentalene as molecular scaffolds and modify these *via* π-extension and nitrogen insertion to generate a set of twelve candidate molecules. We present results on the stabilities of all relevant tautomers in ground and excited states along with absorption/emission energies and intensities. In addition, we highlight the underlying electronic structure in terms of aromaticity as well as orbital transitions. This study suggests that ESA generation *via* a proton transfer is indeed a viable route to unlock powerful new photochemistry.

## 2 Results and discussion

We performed computations on CBD and pentalene derivatives and the results of these will be discussed in the following two sections. Subsequently a brief analysis of the accessible ground state tautomers is shown. Aside from CBD and pentalene derivatives, we have attempted some computations on cyclooctatetraenes, which are discussed in Sec. 3 of the ESI.†

### 2.1 Cyclobutadiene (CBD) derivatives

**Fig. 2:**
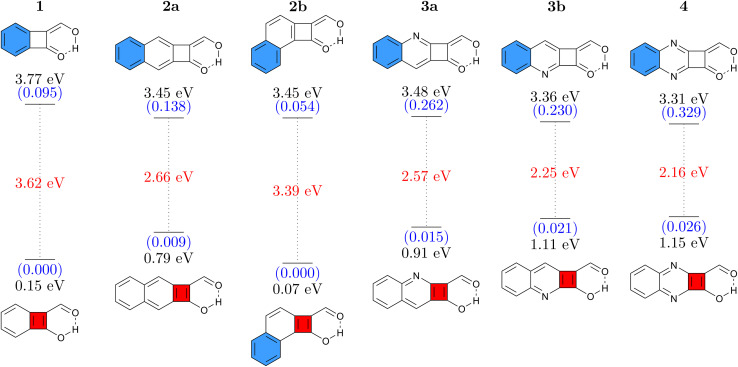
Vertical absorption energies at the ADC(2)/def2-TZVPP level in the Q form (top) and emission energies in the A form (bottom) for CBD derivatives studied. Oscillator strengths (in parentheses, blue) and Stokes shifts (red) are given as well.

Six different cyclobutadiene derivatives were investigated, as presented in Fig. 2. The Q forms, stable in the ground state, are shown at the top and the A forms at the bottom where blue and red filled rings represent [4*n* + 2] and [4*n*] π-electron circuits, respectively. Vertical absorption energies are shown at the top, emission energies at the bottom, and oscillator strengths are given in parentheses. The Stokes shift, computed as the difference between absorption and emission, is shown in red.

The simple CBD based structure shown in Fig. 1 proved to be unsuitable because of its highly unstable ground-state (considering any tautomer) resulting from strong antiaromaticity. Therefore, we start the discussion with 1 (shown in Fig. 2, left), which is based on benzocyclobutadiene. This molecule absorbs at 3.77 eV when computed at the ADC(2)/def2-TZVPP level of theory. Its formal vertical emission energy is computed at 0.15 eV meaning that, according to the energy gap law, it is predicted to deactivate without emission. The difference between absorption and formal emission (3.62 eV) is striking highlighting the pronounced structural rearrangement this molecule undergoes after a photon is absorbed. 1 might act as a photoprotector or photothermal agent; it filters out UV rays with an estimated absorption maximum at 329 nm and would be able to downconvert this to thermal energy *via* an ultrafast proton transfer. However, if the target is luminescence, then it is necessary to increase the S_1_/S_0_ energy gap of the A form, which essentially means reducing ESA and/or reducing ground-state antiaromaticity. For this purpose, we attempt two strategies: π-extension by adding a fused benzene ring and N-insertion at different positions. Combined, these yield the five molecules 2a, 2b, 3a, 3b, and 4. We find that, on the whole, these modifications have the desired effect of lowering the absorption energy and increasing the emission energy. In addition, molecules 2–4 also possess significantly enhanced oscillator strengths making them more potent potential emitters. The exception to these trends is 2b, which has an even lower formal emission energy than 1. We can explain this difference *via* the additional kink in the molecular structure, which provides the possibility of simultaneously having a Hückel aromatic sextet and a Baird aromatic quartet. In line with ref. 59 and 60 this provides particular stabilisation to the excited state, hence lowering its energy.

Remarkably, all molecules shown in Fig. 2 have their absorption energies in the ultraviolet (UV) region while emission is in the infrared (IR) region of the spectrum. Fig. 2 also shows that the oscillator strengths can be effectively tuned *via* π-extension and heteroatom substitution. These properties combined render these molecules interesting candidates for novel emitters with extremely large Stokes shifts.

For comparison and to estimate the robustness of these results, we present the analogous data computed at the M06-2X/def2-TZVP level in Fig. S3.† At the M06-2X level the energies are consistently slightly higher (by 0.2–0.5 eV). But, importantly, the remarkable Stokes shifts of all these molecules are reproduced very well, highlighting that the findings presented here are robust with respect to the level of theory chosen.

**Fig. 3:**
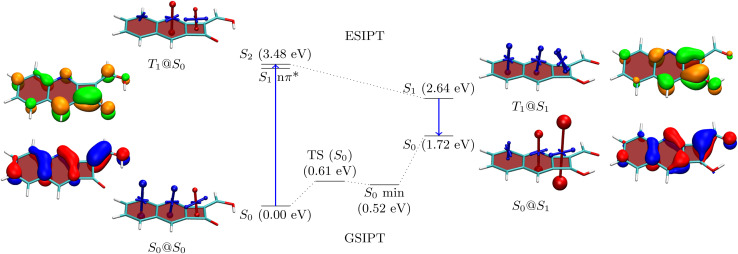
Analysis of proton transfer induced ESA gain for molecule 3a. The quinoidal (Q) and (anti)aromatic (A) forms are shown to the left and right, respectively. Energies are given in parentheses. Next to the energies VIST plots as well as the NTOs (blue/red for hole and orange/green for electron) are shown.

To understand the properties of these molecules in more detail, we consider the example of 3a, presenting an analysis in Fig. 3. The Q form is shown on the left, and the A form on the right. The first main absorption of the Q form is to the S_2_ (ππ*) state lying at 3.48 eV, with a dark nπ* state below it. The associated natural transition orbitals (NTOs) are presented to the left in Fig. 3. These are fairly well delocalised over the whole molecule highlighting the impact of π-extension on its electronic structure.

As a next step, to represent the aromaticity of this system, we use the visualisation of shielding tensors (VIST) method developed by some of us,^61^ providing a visual representation of the nucleus independent chemical shift (NICS).^62^ It is the power of NICS that, when computed at the center of a ring, it intrinsically allows discrimination between aromaticity (NICS < 0) and antiaromaticity (NICS > 0) and that this relation also applies to excited states.^6,7^ Accordingly, within our VIST method shielded (aromatic) components are shown in blue and deshielded (antiaromatic) components are shown in red. Conversely, we also note that computing a single NICS value per ring, as done here, provides only a very condensed view of the underlying currents^63,64^ and, even more, that ring currents do not necessarily go along with energetic stabilisation.^65^ Therefore, to provide an alternative viewpoint, we have also computed three electronic descriptors: the aromatic fluctuation index (FLU),^66^ and the two multicenter bond indices MCI^67^ and *I*_ring_.^68^

Starting with the ground-state shielding tensors evaluated at the ground-state geometry (S_0_@S_0_), we find pronounced shielded components (−27.9/−19.7 ppm) for the six-membered rings in line with their expected aromaticity. For the quinoidal four-membered ring, we only find a minor deshielded contribution (+12.0 ppm), most of which probably derives from ring currents in the neighbouring rings rather than actually indicating antiaromaticity.

We are, next, interested in illustrating the changes in ES(A)A occurring after vertical absorption. Computing magnetic properties for singlet excited states is not routinely possible and, therefore, we compute VIST plots for the T_1_ instead, noting that it possesses ππ* character similar to S_2_. The T_1_@S_0_ VIST plot is shown to the upper left highlighting the pronounced changes in aromaticity characteristics found upon absorption. After absorption the pronounced shielded (blue) tensor components disappear. By contrast a deshielded (+19.7 ppm) tensor appears on the central benzene ring consistent with ESAA on that ring.

Barrierless ESIPT from the Q to the A form strongly lowers the energy of the excited state (from 3.48 to 2.64 eV). This process is accompanied by a pronounced change in magnetic properties yielding only shielded (blue) VIST contributions, consistent with aromaticity, as highlighted on the upper right of Fig. 3 (T_1_@S_1_). Vertical emission to the ground state, finally, entails a dramatic change of magnetic properties with an extremely strong deshielded (+96.5 ppm) component on the CBD ring indicating strong antiaromaticity at this position. The antiaromaticity destabilizes the A form at this geometry, which lies 1.72 eV above the Q-form S_0_ minimum. Optimization of the geometry yields an S_0_ minimum of the A form at 0.52 eV. This can tautomerize to the initial Q form *via* ground-state intramolecular proton transfer (GSIPT) through a small barrier.

Table S2† presents the FLU, *I*_ring_ and MCI analysis for 3a in its S_0_@S_0_ and T_1_@S_1_ states. The S_0_@S_0_ FLU values are 0.010/0.023/0.101 for the three rings, read from left to right. This indicates aromaticity for the six-membered rings and antiaromaticity for the four-membered ring in line with the VIST plot. Upon excitation to the T_1_@S_1_ state we find that the FLU value of the four-membered ring notably reduces (to 0.054). Even more, when the last two rings are considered together as a benzo-CBD unit, we obtain a FLU value of 0.024, providing a clear signature of ESA. ESA is also reflected in enhanced I_ring_ and MCI values after excitation. Following ref. 10 and 13, we also compute ΔFLU_*αβ*_/FLU ratios, that is, we compute the difference between the FLU values for *α* and *β* spins, normalised by the overall FLU. This value is per construction zero for closed-shell Hückel aromatic molecules whereas a significant deviation from zero is seen as a signature of Baird aromaticity.^10^ Viewing the A form of 3a in the T_1_ state, we find that the ΔFLU_*αβ*_/FLU ratios for the three rings, shown from left to right in Fig. 3, are −0.78/0.14/−0.22. The low values on the last two rings run counter to the assignment of Baird aromaticity, suggesting that *α*- and *β*-spin delocalisation contributes to an equal extent. A more detailed analysis of these effects is out of the scope of this work. Nonetheless, we can summarise that the electronic indices support the assignment of ESA but that the overall picture is fairly complicated with all the rings contributing in intricate ways.

The NTOs for emission are presented to the far right in Fig. 3. These possess a similar shape to the NTOs for absorption, with the difference that they are more focussed around the CBD ring, highlighting that the largest electronic structure changes happen there. Indeed, the hole and electron NTOs both possess one nodal plane on the CBD unit, which fits with the antiaromatic-to-aromatic transition on an isolated CBD ring when excited from S_0_ to S_1_.^60^ It is interesting to compare these NTOs to the ones of 2b, as shown in Table S16.†2b, which is the molecule with the lowest formal emission energy, is characterised by NTOs strongly localised on the CBD unit in line with enhanced ESA and ground-state anti-aromaticity.

Finally, considering synthetic accessibility, we note that molecules closely related to 1 have been accessed *via* pyrolysis or photolysis of 2-diazo-1,3-indandione.^69,70^ More generally, we note that benzocyclobutenedione photochemistry is studied quite widely.^71,72^ Furthermore, functionalised biphenylenes, related to 2a but with the CBD between the two benzene rings, have been synthesised for use as photoswitches.^22^ Combined, this suggests that synthesis of the CBD derivatives studied within this work or related molecules with analogous functionality is indeed a promising new avenue.

### 2.2 Pentalene derivatives

**Fig. 4:**
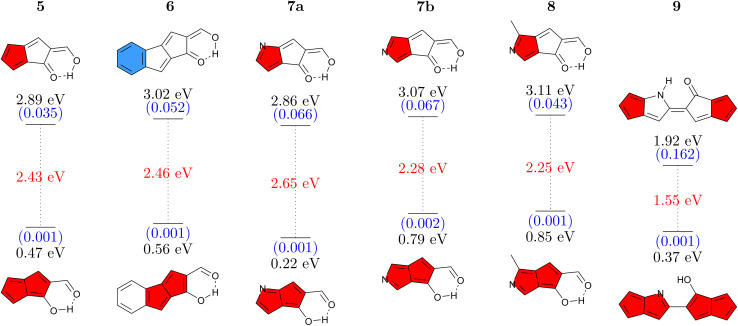
Vertical absorption energies at the ADC(2)/def2-TZVPP level in the Q form (top) and emission energies in the A form (bottom) for pentalene derivatives studied. Oscillator strengths (in parentheses, blue) and Stokes shifts (red) are given as well.

Having studied CBD, we proceed to a second antiaromatic motif, pentalene. Pentalene is formed of two fused five-membered rings, possessing 8 π-electrons in total. In a similar sense as before, we aim at breaking the conjugation *via* a quinoidal structure. Six pentalene derivatives were constructed in this fashion (Fig. 4). The parent molecule 5 absorbs at 2.89 eV and, similarly to the basic CBD derivative 1, has an emission energy below 1 eV along with a low oscillator strength, which together suggests that this molecule would not be emissive. Again, we tune the properties *via* π-extension and N-atom substitution. Fusing a benzene ring, to produce 6 increases the energy gap while also slightly increasing the Stokes shift. The higher absorption energy of 6 can be rationalized by considering that its Q form can be drawn with a resonance structure possessing only a sextet and no quartet.

Continuing with N-atom substituted derivatives, we find that the effect of adding a nitrogen atom crucially depends on where it is positioned. In the case of 7a the N-atom is positioned on an atom with a strong LUMO contribution (see below) and this lowers absorption and emission energies. The opposite is true for 7b, which features raised energies. Even slightly higher gaps are obtained for 8 where an N-atom is placed to stabilize the HOMO while a methyl group is used to destabilize the LUMO. We finish with 9 where two pentalene units are joined together, following ideas from ref. 56. Due to enhanced delocalization, we find that the absorption energy is significantly reduced compared to the others and that the formal emission energy is also very low (0.37 eV). However, it is worth noting that unlike any of the others, the Q form of 9 forms a local minimum on the S_1_ surface, thus providing the possibility for dual emission. Reviewing Fig. 4 as a whole, we find that the energy gaps in this series can be tuned in analogy to the CBD derivatives discussed above. On the whole, the absorption and emission energies of the pentalene derivatives are about 0.5–1.0 eV lower than those of the CBD derivatives. As opposed to the CBD derivatives, we were not able to find any derivative with appreciable oscillator strength for emission (above 0.003).

**Fig. 5:**
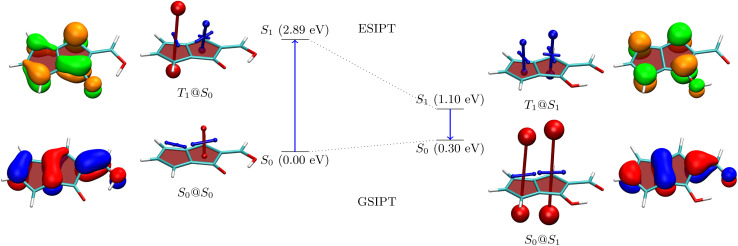
Analysis of proton transfer induced ESA gain for molecule 5 (see caption of Fig. 3 for details).

For a more detailed discussion of the electronic structure properties, we choose 5, as shown in Fig. 5. The ground state of the Q form is seen to be largely non-aromatic with only a slightly deshielded (anti-aromatic) component of +10.7 ppm on one of the five-membered rings. The Q form absorbs at 2.89 eV, which is at the blue edge of the visible spectrum. After absorption a strongly antiaromatic component (+68.9 ppm) is found on the left 5-membered ring. In the S_1_ state the molecule can undergo barrierless ESIPT to the A form lowering its energy by 1.79 eV. This process induces aromaticity as reflected by notable shielding (−28.4 and −15.5 ppm). From there the molecule would progress to the ground state *via* a long wavelength emission of 1550 nm (0.80 eV) or non-radiative decay. After vertical emission, a strongly antiaromatic electron configuration with deshielding on both rings (+127.5 and +103.3 ppm) is obtained. Subsequently GSIPT restores the Q form in the ground state. The electronic aromaticity descriptors computed (see Table S2†) largely support the above assignment. In the present case, a clear signature of Baird aromaticity (ΔFLU_*αβ*_/FLU ≈ −1) is observed for both rings.

Reviewing the orbitals, we find a particularly symmetric shape for the A form (right) resembling those of the parent pentalene. The hole and particle NTOs both have two nodal planes, which fits well with an antiaromatic/aromatic transition,^60^ as discussed above. Furthermore, we find that the HOMO and LUMO reside on mutually exclusive atoms. This fact provides good opportunities for shifting the energies of these orbitals independently as shown in Fig. 4. Conversely, the orbitals also help explain the low oscillator strength of the emitting state. First, low orbital overlap is obtained due to the fact that the HOMO and LUMO reside on alternating atoms. Second, Fig. 5 (right) highlights that the S_1_ state derives from the Laporte forbidden state of *gerade* symmetry in pentalene. Both factors combined explain the challenges we encountered here in designing pentalene derivatives with strong oscillator strengths.

### 2.3 Ground-state tautomers

**Fig. 6:**
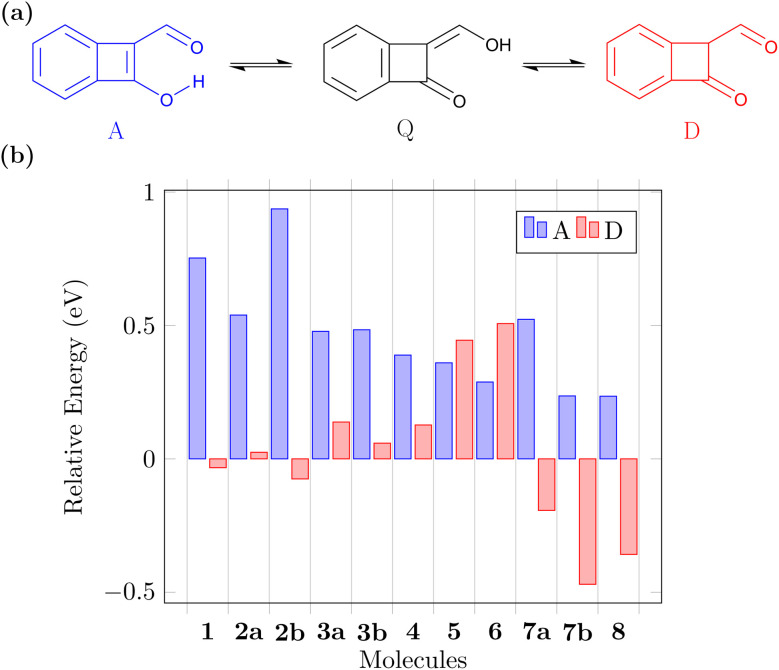
Comparison of ground-state tautomer energies: (a) possible interconversion between the (anti)aromatic (A), quinoidal (Q), and diketo (D) forms; (b) S_0_ energy of the A and D forms relative to the Q form determined for molecules 1–8.

Before concluding, we want to address another relevant question. The suggested ESIPT process is only possible if the proposed Q form is indeed the lowest energy tautomer in the ground state. Note that, aside from the A and Q forms, these molecules also possess various possible diketo (D) forms that may come into play (see Fig. 6a for an example) and we will now investigate the potential involvement of these. Fig. 6b presents the energies of the A and lowest energy D form for each molecule relative to that of the respective Q form (more details given in Fig. S2†). We find that for the CBD based molecules 1, 2a, and 2b the energies of the Q and D forms are similar. Introduction of the nitrogen atoms notably turns this balance in favour of the Q form, which is the most stable ground state tautomer for 3a, 3b, and 4. The pentalene derivatives 5 and 6 retain high-energy D forms noting that these still possess one antiaromatic ring. Viewing the pentalene derivatives with a nitrogen atom (7a, 7b, and 8), we find that the most stable tautomer is the one where the proton is attached to nitrogen forming a pyrrole ring, meaning that these molecules would not be undergoing ESIPT. However, we want to highlight that this problem can be avoided by indeed using the N-atom as the hydrogen bond donor, as exemplified in 9. In summary, we find that the D forms cannot be ignored but also that ample opportunity exists to stabilise the Q form when designing new related molecules.

## 3 Conclusions and outlook

In this work we have highlighted the feasibility of aromaticity generating ESIPT as a new type of photophysical process. Two classes of molecules (CBD and pentalene derivatives) were designed and investigated computationally. We presented results on the relevant energetic properties and provided detailed insight into the electronic structure *via* aromaticity descriptors and NTO plots. The most notable properties of the molecules studied were dramatic Stokes shifts, in excess of 2 eV, meaning that these molecules could act as UV absorbers and IR emitters. The dramatic change in energy gap upon excited-state relaxation was rationalised by analysing the pronounced changes in their underlying electronic structure. The most promising candidates for ultra-large Stokes shift emitters were the π-extended and N substituted CBD derivatives 3a and 4.

More generally, we highlighted several prerequisites for successfully designing appropriate chromophores. Most notably, in the smaller chromophores studied, excited-state relaxation is so pronounced that no emission is expected due to the energy gap law. Such chromophores could be used for photoprotection or photothermal applications, but the design of emissive chromophores necessitates a careful tuning of ESA gain. Furthermore, we have outlined strategies for increasing oscillator strengths *via* heteroatom substitution. Finally, we highlighted the importance of different possible ground-state tautomers in the systems studied.

In summary, we believe that proton transfer induced excited-state aromaticity gain, when integrated into a rational design process, provides a powerful route for unlocking new photochemistry.

## 4 Computational details

All geometry optimizations were performed at the M06-2X/def2-TZVP level of theory using density functional theory (DFT) and time-dependent DFT (TDDFT) within the Tamm–Dancoff approximation for ground and excited-state structures, respectively.^73–75^ The nature of all structures was confirmed by frequency calculations. Vertical excitation energies were computed using the algebraic diagrammatic construction to second order, ADC(2), within the resolution-of-the-identity approximation along with the def2-TZVPP basis set.^76–78^ DFT computations were performed using Q-Chem^79^ and ADC(2) computations using TURBOMOLE.^80^ To estimate the accuracy of these computational methods, we have computed the absorption and emission energies of a few related molecules known from the literature;^71,81–84^ the results highlight that both methods are suitable with ADC(2) being particularly reliable.

Nucleus-independent chemical shift (NICS) values^62^ were calculated at the PBE0/def2-SVP level^74,85^ as implemented in Gaussian-09.^86^ The aromaticity of ground and excited states was analysed using the VIST (visualisation of chemical shielding tensors) technique^61^ as implemented in the TheoDORE 3.1 package.^87^ Ground-state NICS were computing using the restricted Kohn–Sham (RKS) method. Considering that singlet excited-state NICS are not routinely available, we compute NICS values for the T_1_*via* the unrestricted Kohn–Sham (UKS) method verifying that the orbitals involved are the same as for the S_1_. All molecular graphics were generated using VMD.^88^ Natural transition orbitals (NTOs) were obtained from M06-2X/def2-TZVP computations.^89–91^

The aromatic fluctuation index (FLU)^66^ as well as multicenter bond indices MCI^67^ and *I*_ring_^68^ were computed for molecules 3a and 5 with the AIMALL^92^ and ESI-3D^93^ programs. Orbitals for these calculations were obtained *via* RKS/UKS for S_0_/T_1_ in analogy to the NICS calculations.

## Data availability

Molecular geometries, input and output files of all relevant computations are presented *via* a separate repository (https://doi.org/10.17028/rd.lboro.26298796).

## Author contributions

D. Xing: investigation, visualization, writing – original draft; F. Glöcklhofer: writing – review and editing; F. Plasser: conceptualization, methodology, supervision, writing – original draft.

## Conflicts of interest

There are no conflicts to declare.

## Supplementary Material

SC-OLF-D4SC04692G-s001
