# Supplementary material for: Proton transfer induced excited-state aromaticity gain for chromophores with maximal Stokes shifts
Source: Chem Sci. 2024 Oct 2;15(43):17918–26. doi: 10.1039/d4sc04692g (PMC11463706; doi:10.1039/d4sc04692g)
Supplement: SC-015-D4SC04692G-s001 [file SC-015-D4SC04692G-s001.pdf]

Supplementary Information (SI) for Chemical Science.
This journal is © The Royal Society of Chemistry 2024

Supporting Information

Proton transfer induced excited-state aromaticity gain for chromophores with maximal Stokes shifts

Dong Xing, Florian Glöcklhofer, and Felix Plasser

E-mail: f.plasser@lboro.ac.uk

Phone: +44 1509 226946

Table of contents

1. Aromaticity Analysis 2

2. Natural transition orbitals (NTOs) 15

3. COT derivatives 27

4. Ground-state tautomers 29

5. Further computational and experimental results 30

6. References 32

1. Aromaticity Analysis

Nucleus-independent chemical shifts (NICS)[1] were calculated at PBE0/def2-SVP level[2,3] using gauge including atomic orbitals as implemented in Gaussian 09[4]. NICS tensors were represented graphically using the visualization of chemical shielding tensors (VIST)[5] method as implemented in TheoDORE[6] and using VMD[7] as a graphical interface. Note that all the presented Q forms are optimized in the S_0 state and all A forms in the S_1 state. The aromatic fluctuation index (FLU)[8], multicenter bond indices MCI[9] and Iring[10] were computed for molecules 3a and 5 with the AIMALL[11] added and ESI-3D[12] programs.

Table S1. The out-of-plane component of NICS value (ppm) at the ring with carbonyl or hydroxyl groups (Ring 1) and further fused ring(s) (Ring 2, 3 and 4) for Molecules **1-9**. (Values smaller than 5 ppm are labeled as "small")

Molecule	State	Ring 1	Ring 2	Ring 3	Ring 4
**1**	Q(S_0)	13	-23		
	Q(T_1)	39.2	31.3		
	A(T_1)	-12	-14		
	A(S_0)	378.9	152.1		
**2a**	Q(S_0)	13.2	-22.1	-27.7	
	Q(T_1)	11.2	32.8	small	
	A(T_1)	-12.4	-14.3	-18.3	
	A(S_0)	106.9	33.9	small	
**2b**	Q(S_0)	14.9	-23.3	-27.3	
	Q(T_1)	25.3	64.5	19.7	
	A(T_1)	-13.1	-7.6	42.9	
	A(S_0)	499.8	126.5	-27.9	
**3a**	Q(S_0)	12	-19.7	-27.9	
	Q(T_1)	8.8	19.7	small	
	A(T_1)	-10.9	-15.6	-17.6	
	A(S_0)	96.5	33.2	5.5	
**3b**	Q(S_0)	12.1	-21.9	-28.1	

	$Q(T_1)$	9.4	25.2	small	
	$A(T_1)$	-12.1	-12.3	-19.4	
	$A(S_0)$	94.5	24.1	-8.7	
**4**	$Q(S_0)$	10.5	-19.7	-28.2	
	$Q(T_1)$	6.3	18.5	small	
	$A(T_1)$	-10.3	-13.3	-17.7	
	$A(S_0)$	87.2	27.2	-8	
**5**	$Q(S_0)$	10.7	small		
	$Q(T_1)$	-17	68.9		
	$A(T_1)$	-28.4	-15.5		
	$A(S_0)$	127.5	103.3		
**6**	$Q(S_0)$	9.8	small	23.2	
	$Q(T_1)$	small	32.4	small	
	$A(T_1)$	-23.3	small	-19.1	
	$A(S_0)$	114.9	73.7	15.6	
**7a**	$Q(S_0)$	11.7	5.7		
	$Q(T_1)$	small	19.5		
	$A(T_1)$	-24.4	-14.5		
	$A(S_0)$	230.1	157		
**7b**	$Q(S_0)$	9.1	small		
	$Q(T_1)$	small	17.8		
	$A(T_1)$	-21	-21.9		
	$A(S_0)$	65.4	56		
**8**	$Q(S_0)$	9.9	small		
	$Q(T_1)$	small	27.7		
	$A(T_1)$	-25.9	-20.5		
	$E(S_0)$	77.2	61.1		
**9**	$Q(S_0)$	24.2	8.2	10.7	small
	$Q(T_1)$	17.3	small	15.7	5
	$A(T_1)$	23.3	11.5	small	small
	$A(S_0)$	49.5	27.7	152	121.7

Table S2. Aromaticity descriptors for S_0 and T_1 states of molecules **3a** and **5**.[a]

		FLU[b]	I_{ring}	MCI	FLU (α/β)	$\Delta FLU_{\alpha\beta}/FLU$
3a Q(S_0)	Ring1 (C_6)	0.010	0.029	0.040		
	Ring2 (C_6)	0.023	0.019	0.025		
	Ring3 (C_4)	0.101	0.013	0.014		
	Ring2+3 (C_8)	0.055	0.001	0.001		
3a A(T_1)	Ring1 (C_6)	0.023	0.018	0.024	0.016/0.034	-0.783
	Ring2 (C_6)	0.029	0.010	0.011	0.036/0.032	0.138
	Ring3 (C_4)	0.054	0.024	0.044	0.056/0.068	-0.222
	Ring2+3 (C_8)	0.024	0.002	0.006	0.031/0.029	0.083
5 Q(S_0)	Ring1 (C_5)	0.033	0.021	0.017		
	Ring2 (C_5)	0.069	0.008	0.007		
	Ring 1+2 (C_8)	0.048	0.002	0.001		
5 A(T_1)	Ring1 (C_5)	0.018	0.013	0.013	0.012/0.032	-1.111
	Ring2 (C_5)	0.028	0.014	0.015	0.017/0.045	-1.000
	Ring1+2 (C_8)	0.010	0.004	0.016	0.006/0.019	-1.300

[a] Ring1, Ring2, Ring3 are arranged from left to right in Figures 3 and 5 of the main article. Ring1+2 refers to the perimeter of Ring1 and Ring2 combined, etc.
[b] Values of FLU < 0.030 and FLU > 0.060 are highlighted in blue and red, respectively, to indicate aromaticity and antiaromaticity.

Table S3. VIST plots for Q and A form of molecule **1** in S_0 and T_1 states.

**1** Q (S_0) 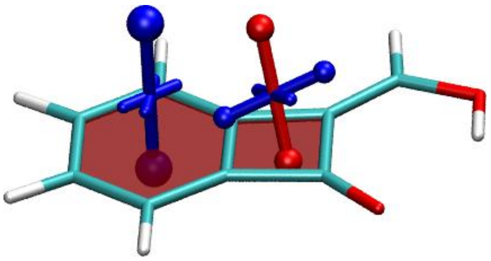	**1** Q (T_1) 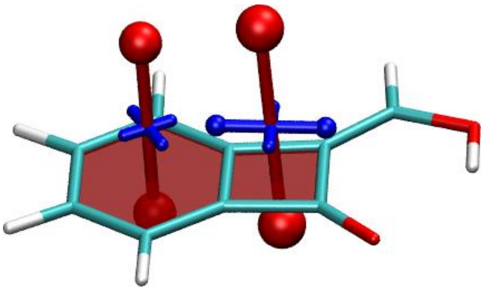
**1** A (S_0) 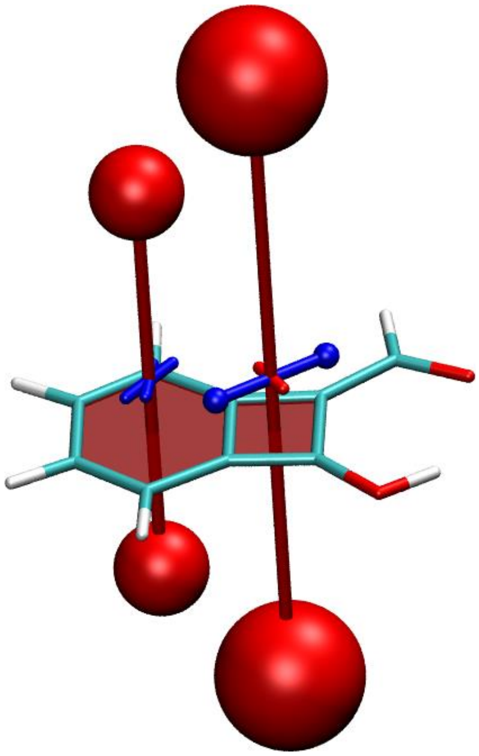	**1** A (T_1) 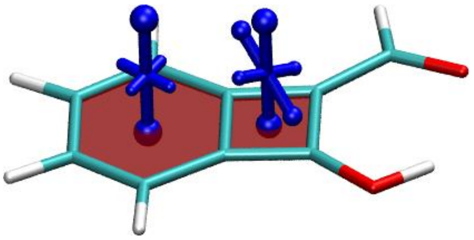

Table S4. VIST plots for Q and A form of molecule **2a** in S_0 and T_1 states.

**2a** Q (S_0)<br>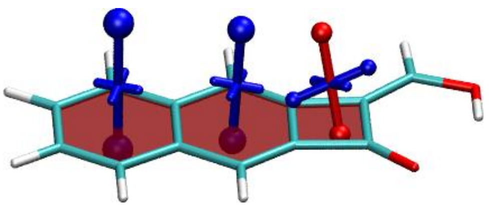	**2a** Q (T_1)<br>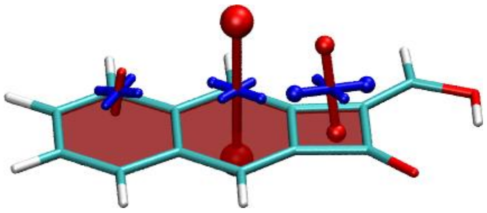
**2a** A (S_0)<br>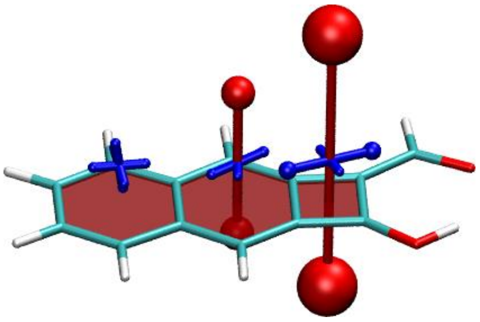	**2a** A (T_1)<br>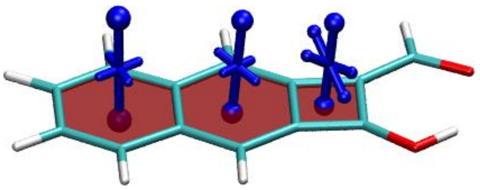

Table S5. VIST plots for Q and A form of molecule **2b** in S_0 and T_1 states.

**2b** Q (S_0)	**2b** Q (T_1)
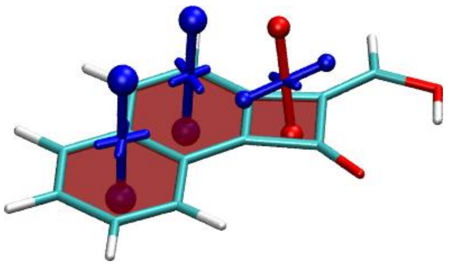	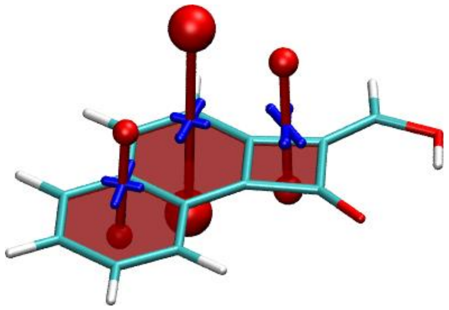
**2b** A (S_0)	**2b** A (T_1)
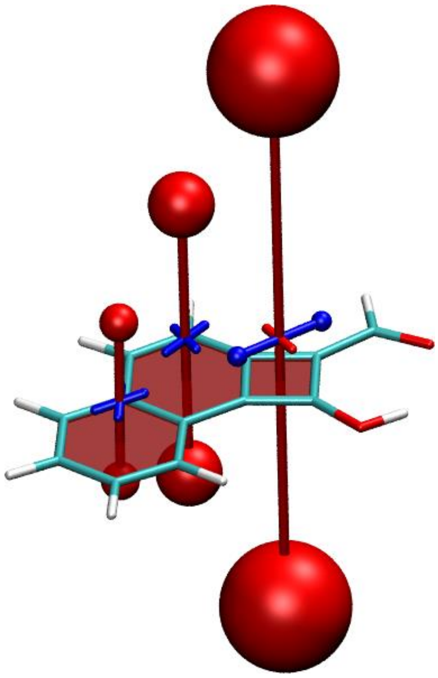	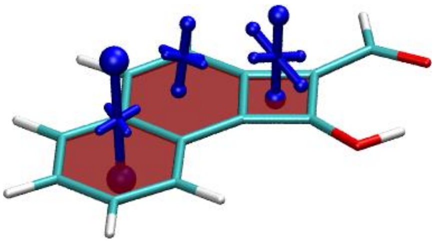

Table S6. VIST plots for Q and A form of molecule **3a** in S_0 and T_1 states.

**3a** Q (S_0)	**3a** Q (T_1)
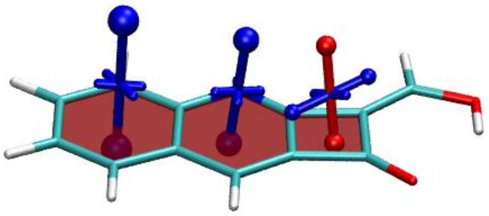	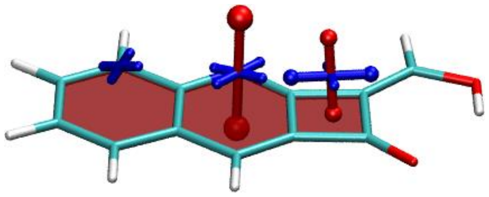
**3a** A (S_0)	**3a** A (T_1)
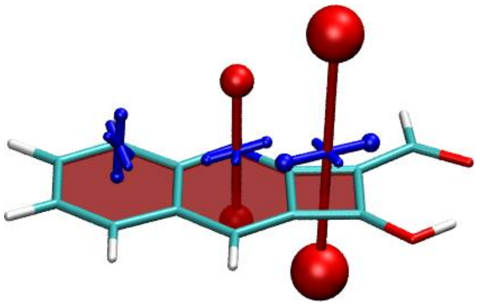	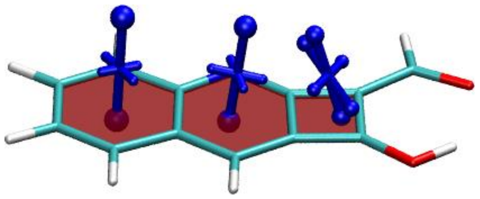

Table S7. VIST plots for Q and A form of molecule **3b** in S_0 and T_1 states.

**3b** Q (S_0)	**3b** Q (T_1)
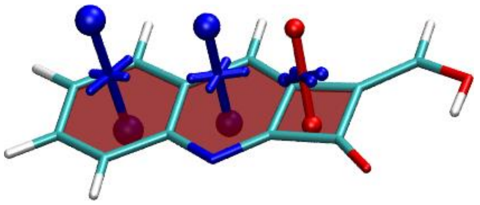	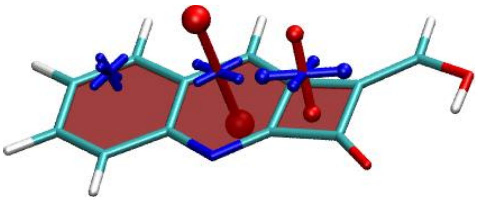
**3b** A (S_0)	**3b** A (T_1)
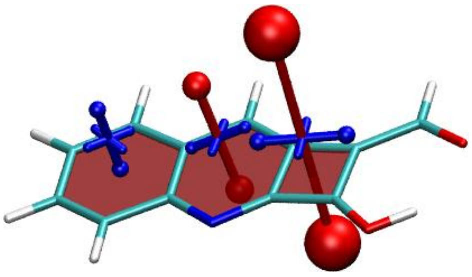	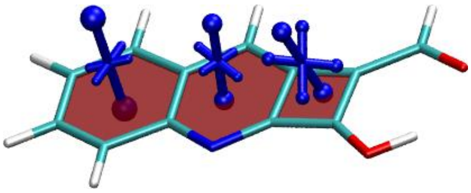

Table S8. VIST plots for Q and A form of molecule **4** in S_0 and T_1 states.

**4** Q (S_0) 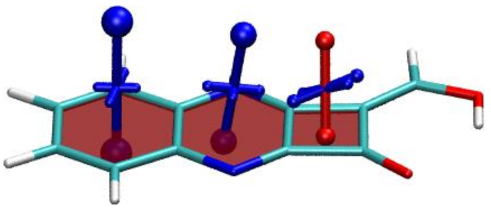	**4** Q (T_1) 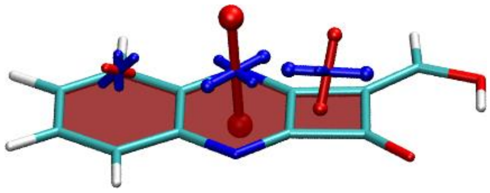
**4** A (S_0) 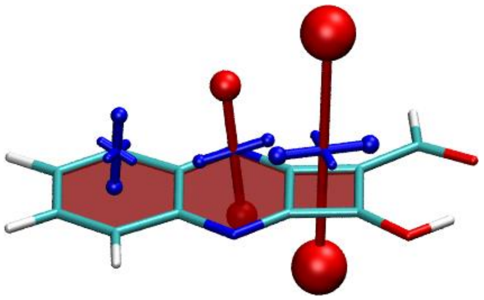	**4** A (T_1) 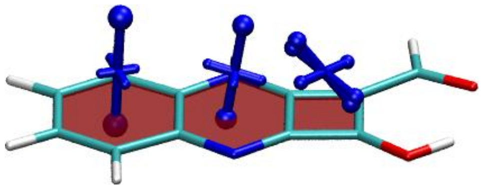

Table S9. VIST plots for Q and A form of molecule **5** in S_0 and T_1 states.

**5** Q (S_0)<br>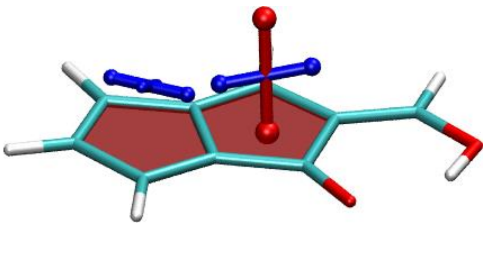	**5** Q (T_1)<br>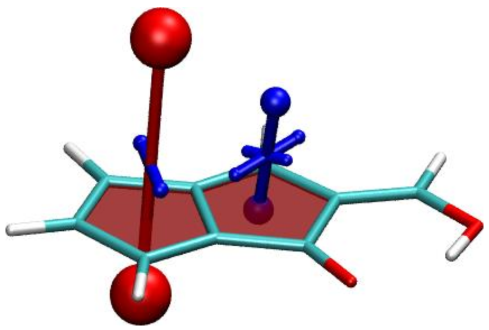
**5** A (S_0)<br>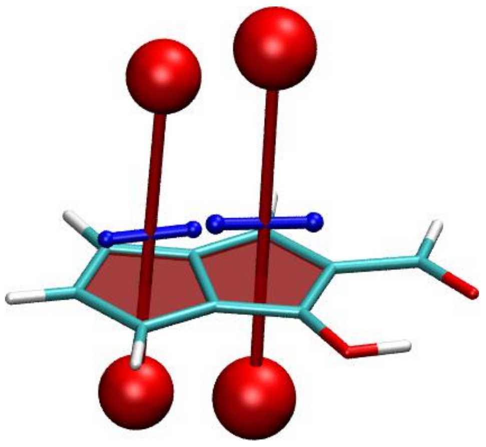	**5** A (T_1)<br>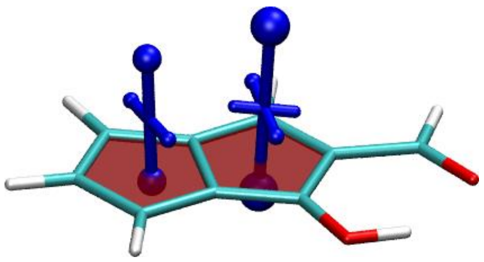

Table S10. VIST plots for Q and A form of molecule **6** in S_0 and T_1 states.

**6** Q (S_0) 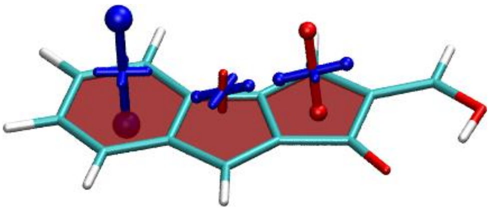	**6** Q (T_1) 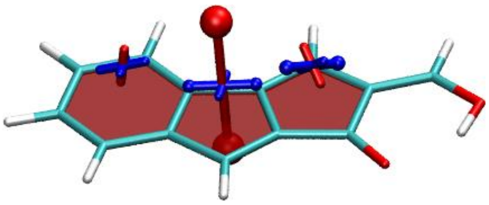
**6** A (S_0) 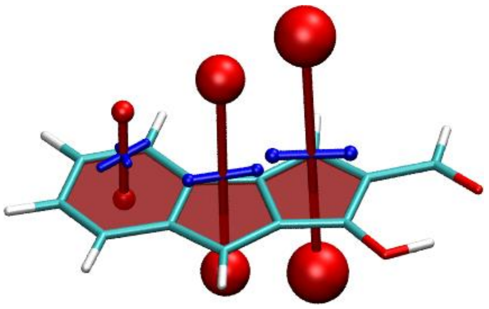	**6** A (T_1) 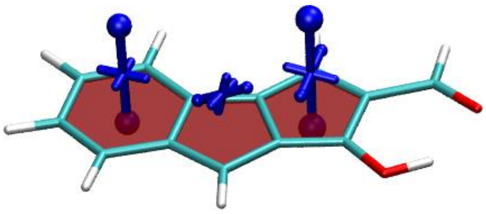

Table S11. VIST plots for Q and A form of molecule **7a** in S_0 and T_1 states.

**7a** Q (S_0)	**7a** Q (T_1)
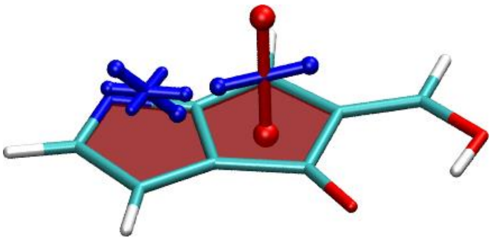	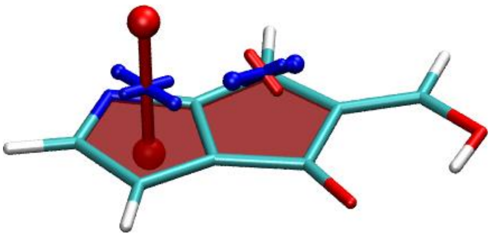
**7a** A (S_0)	**7a** A (T_1)
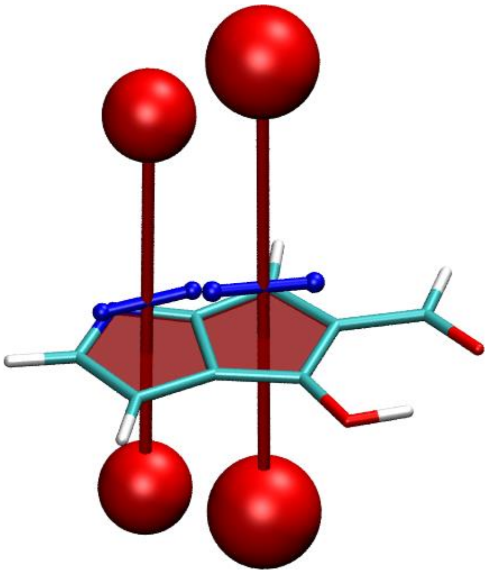	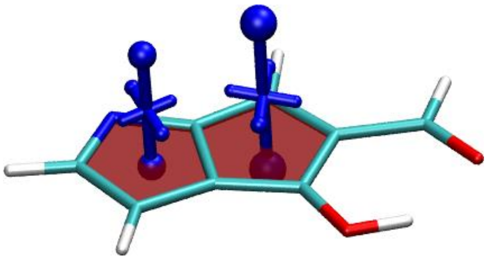

Table S12. VIST plots for Q and A form of molecule **7b** in S_0 and T_1 states.

**7b** Q (S_0)	**7b** Q (T_1)
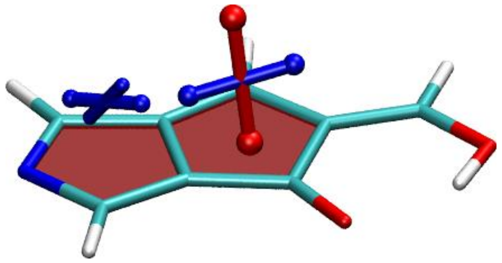	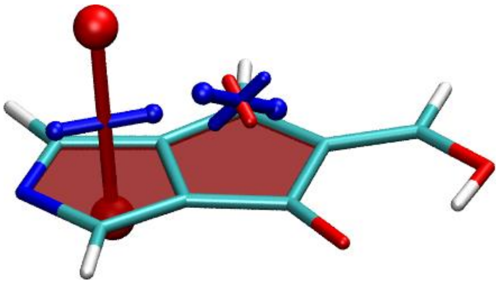
**7b** A (S_0)	**7b** A (T_1)
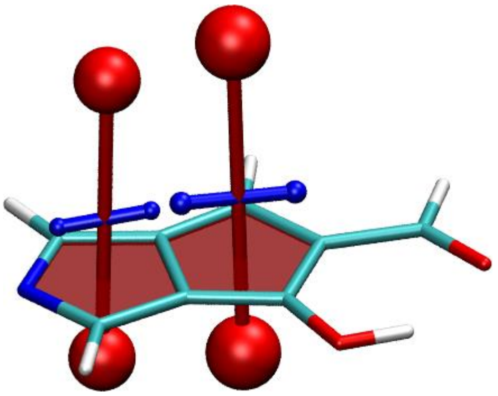	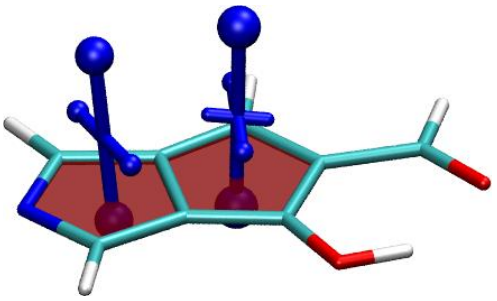

Table S13. VIST plots for Q and A form of molecule **8** in S_0 and T_1 states.

**8** Q (S_0)<br>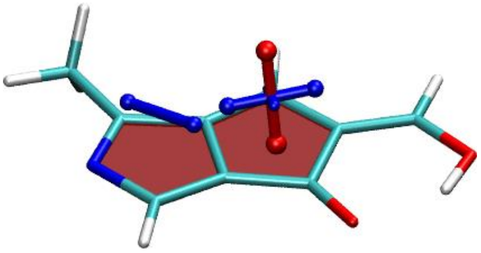	**8** Q (T_1)<br>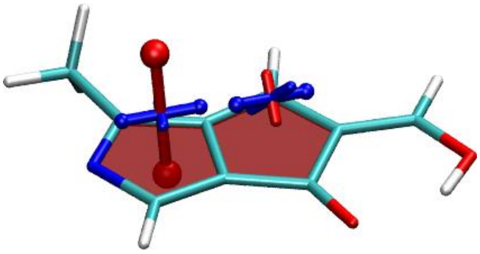
**8** A (S_0)<br>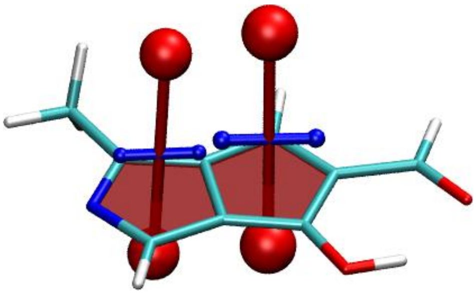	**8** A (T_1)<br>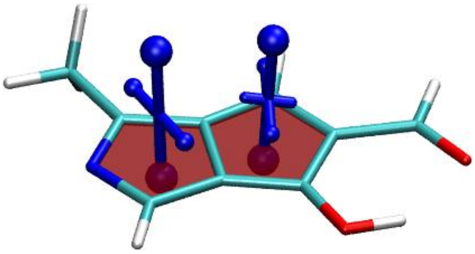

Table S14. VIST plots for Q and A form of molecule **9** in S_0 and T_1 states.

**9** Q (S_0)<br>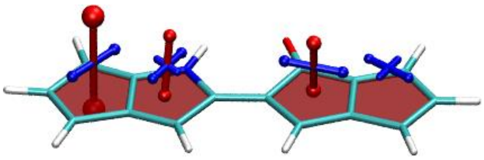	**9** Q (T_1)<br>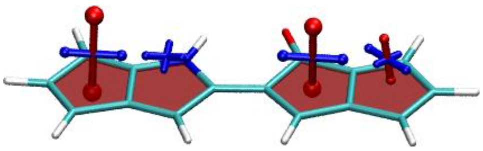
**9** A (S_0)<br>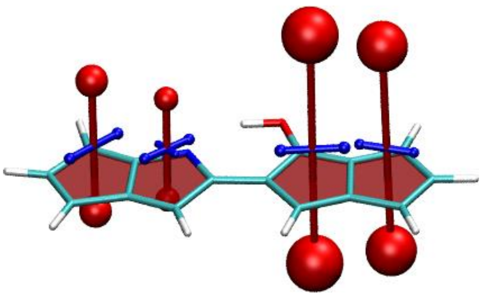	**9** A (T_1)<br>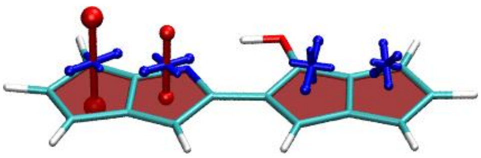

2. Natural transition orbitals (NTOs)

Natural transition orbitals[13] are computed after single point TDDFT calculation at M06-2X[14]/def2-TZVP level and visualized by Jmol. Note that all the NTOs of Q forms presented are at S_0 geometry and A forms are at S_1 geometry.

Table S15. Natural transition orbitals of molecule **1**. (blue/red for hole and orange/green for electron)

**1** Q	**1** Q
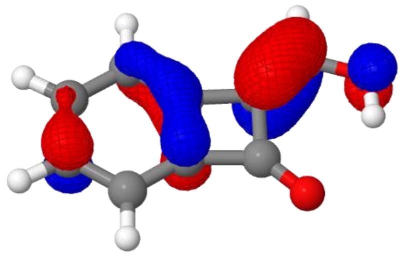	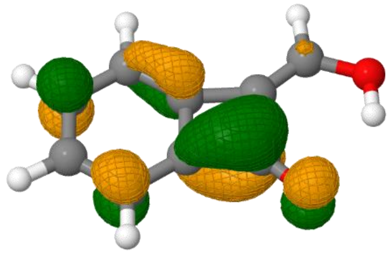
**1** A	**1** A
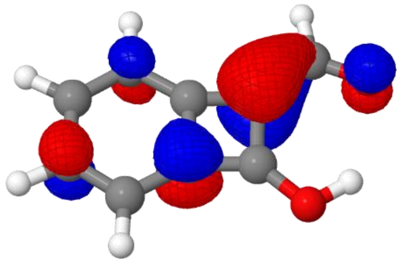	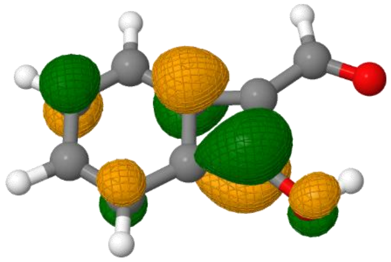

Table S16. Natural transition orbitals of molecule **2a**. (blue/red for hole and orange/green for electron)

**2a** Q<br>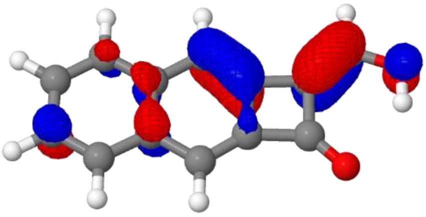	**2a** Q<br>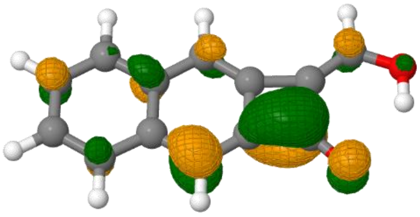
**2a** A<br>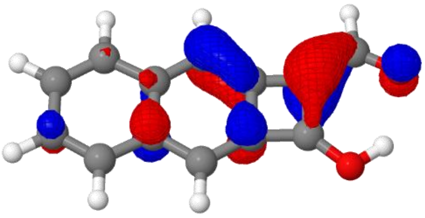	**2a** A<br>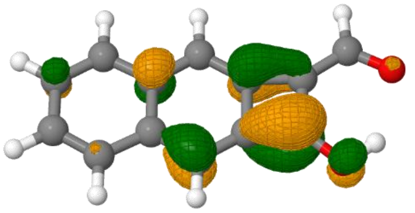

Table S17. Natural transition orbitals of molecule **2b**. (blue/red for hole and orange/green for electron)

**2b** Q	**2b** Q
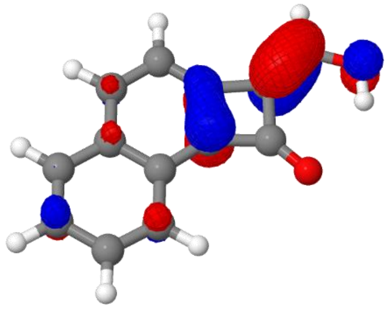	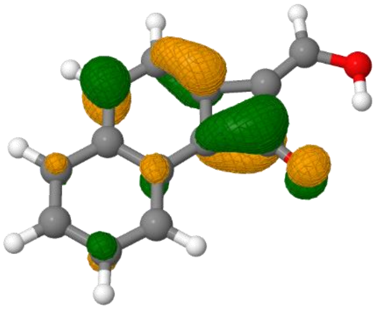
**2b** A	**2b** A
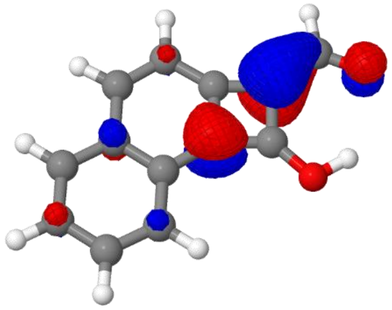	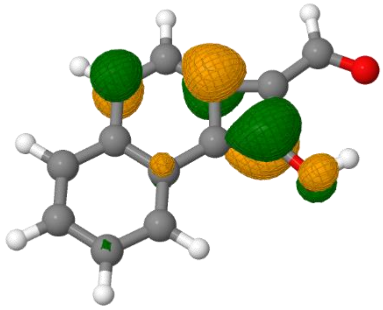

Table S18. Natural transition orbitals of molecule **3a**. (blue/red for hole and orange/green for electron)

**3a** Q	**3a** Q
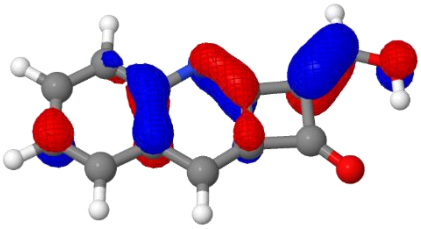	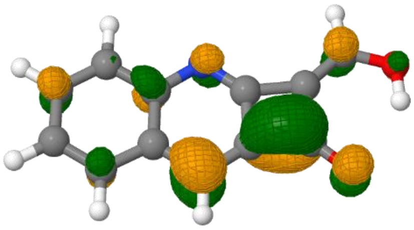
**3a** A	**3a** A
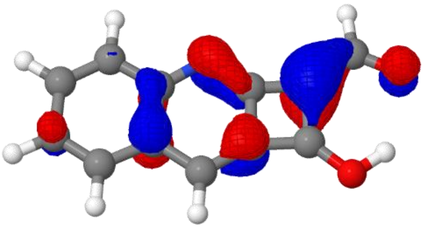	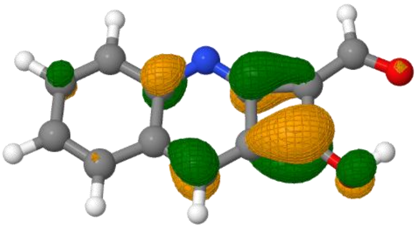

Table S19. Natural transition orbitals of molecule **3b**. (blue/red for hole and orange/green for electron)

**3b** Q	**3b** Q
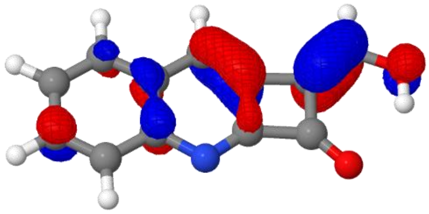	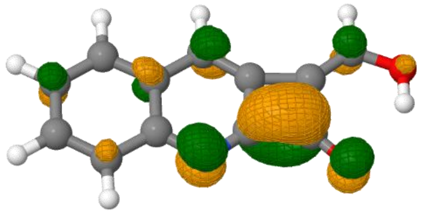
**3b** A	**3b** A
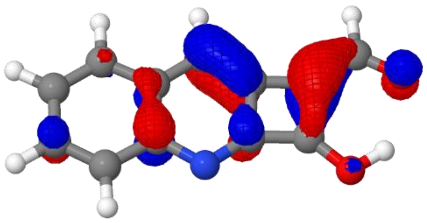	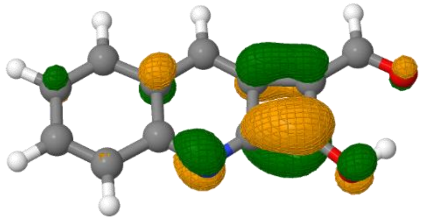

Table S20. Natural transition orbitals of molecule **4**. (blue/red for hole and orange/green for electron)

**4** Q	**4** Q
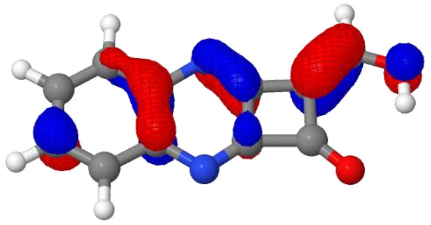	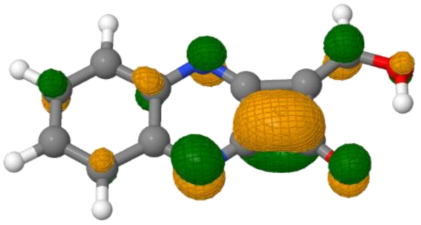
**4** A	**4** Q
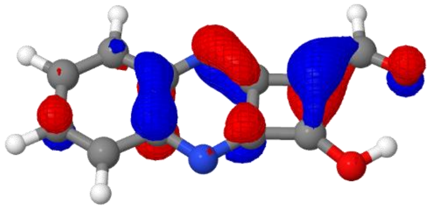	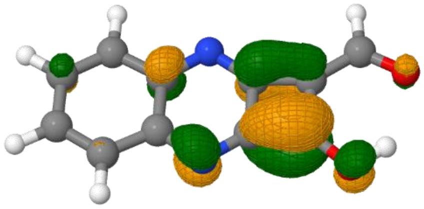

Table S21. Natural transition orbitals of molecule **5**. (blue/red for hole and orange/green for electron)

**5** Q	**5** Q
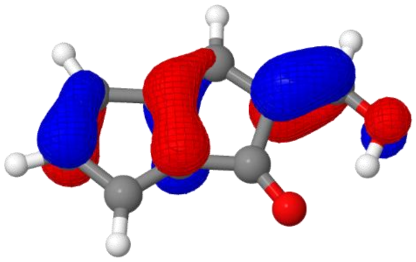	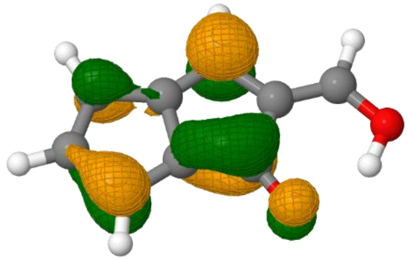
**5** A	**5** A
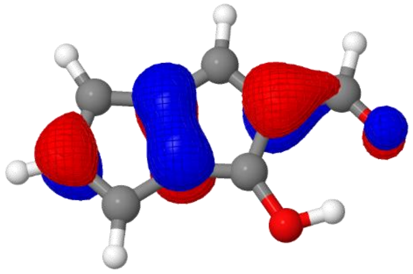	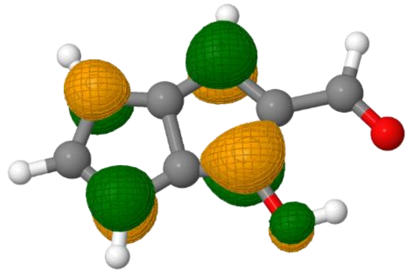

Table S22. Natural transition orbitals of molecule **6**. (blue/red for hole and orange/green for electron)

**6** Q	**6** Q
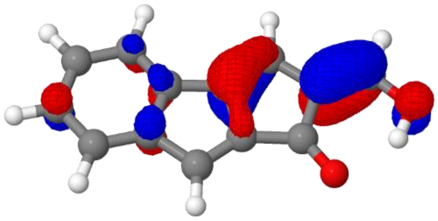	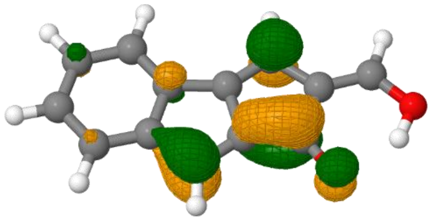
**6** A	**6** A
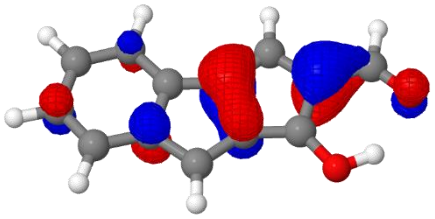	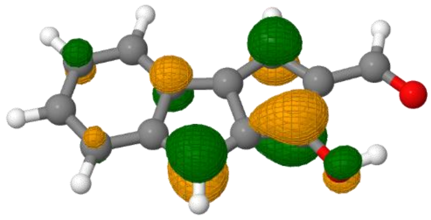

Table S23. Natural transition orbitals of molecule **7a**. (blue/red for hole and orange/green for electron)

**7a** Q	**7a** Q
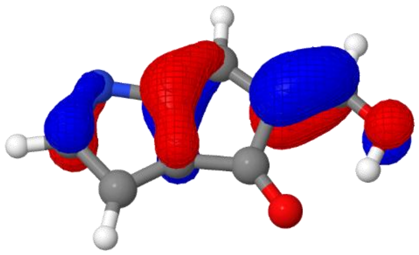	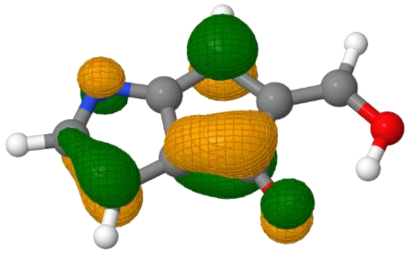
**7a** A	**7a** A
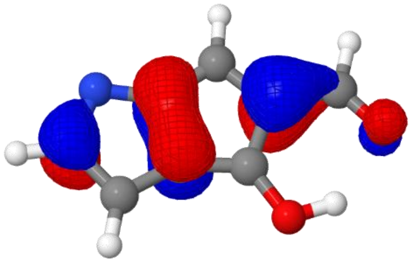	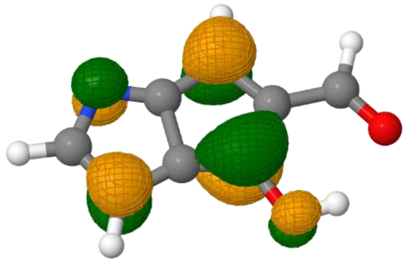

Table S24. Natural transition orbitals of molecule **7b**. (blue/red for hole and orange/green for electron)

**7b** Q	**7b** Q
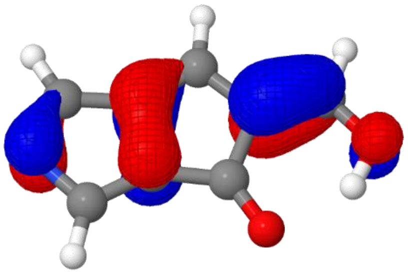	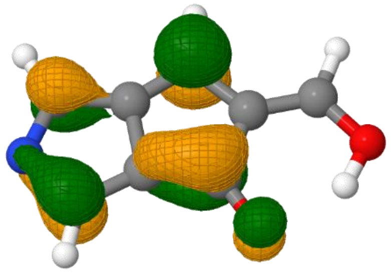
**7b** A	**7b** A
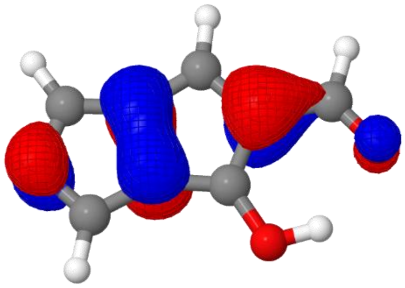	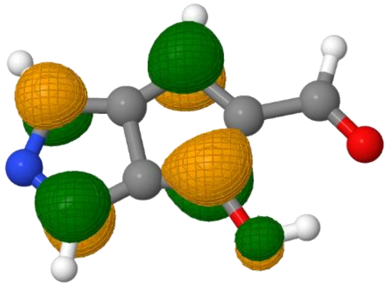

Table S25. Natural transition orbitals of molecule **8**. (blue/red for hole and orange/green for electron)

**8** Q	**8** Q
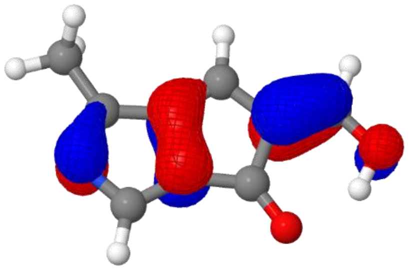	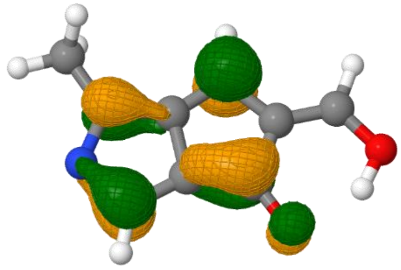
**8** A	**8** A
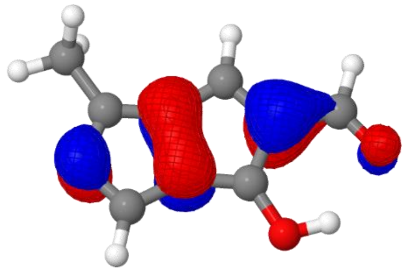	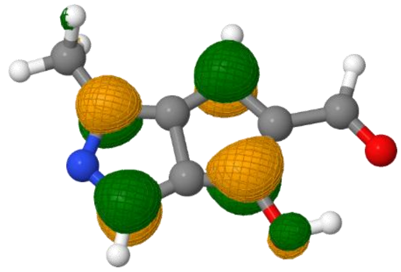

Table S26. Natural transition orbitals of molecule **9**. (blue/red for hole and orange/green for electron)

**9** Q<br>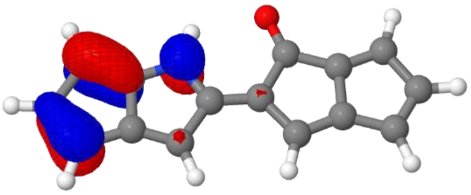	**9** Q<br>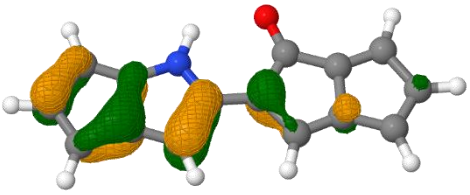
**9** A<br>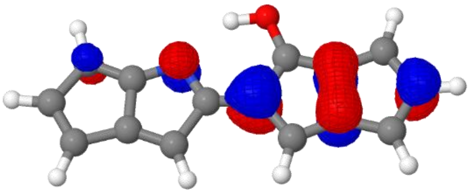	**9** A<br>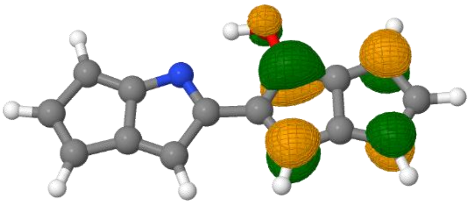

3. COT derivatives

Large difference between results of COT (Cyclooctatetraene) derivatives and the previous two groups of molecules were found. Considering molecules geometries, we note that for all four molecules, the COT ring of Q(S_0) is not planar, whereas the planarity of A(S_1) is higher, but the molecule shows a twisted structure, especially for the COT ring. Figure S1 illustrates the analysis on vertical excitations with COT derivatives. The vertical excitation energies for COT derivatives are similar among each other with only small variations. Even the absorption oscillator strengths for absorption are always small. The additional fused ring gives almost no difference on the photophysical property, and no trends can be observed.

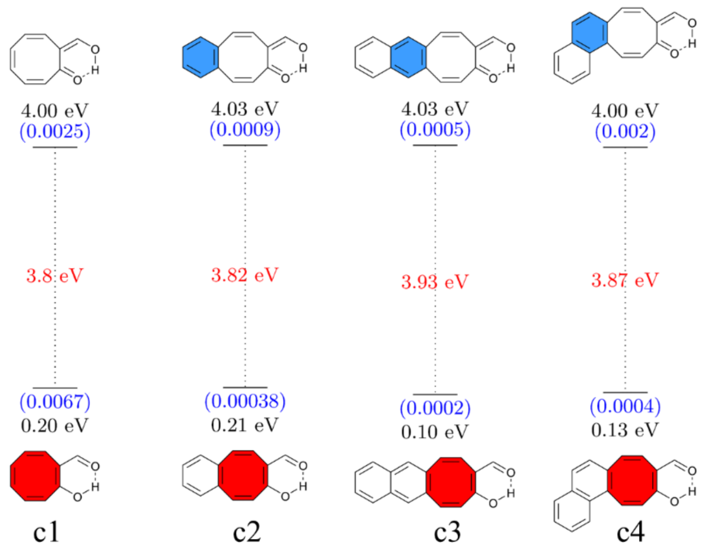

Figure S1. Vertical absorption energies in the Q form (top) and emission energies in the A form (bottom) at the M06-2X/def2-TZVP level for COT derivatives studied. Oscillator strengths (in parentheses, blue) and Stokes shifts (red) are given as well.

COT derivates are characterized by extremely strong deshieding (antiaromaticity) of S_0 at their S_1 (A form) geometries. In line with this finding, the form S_1 emission energies are extremely low.

Viewing the properties of the COT derivatives we do not believe them to be suitable

for optical applications and discuss them no further.

Table S27. the out-of-plane NICS value of the COT ring in molecules c1 - c4

molecules	NICS(0)$_{ZZ}$ of 8-membered ring in S_0@S_1 (ppm)
c1	+737.3024
c2	+711.1718
c3	+5278.2774
c4	+1013.7536

4. Ground-state tautomers

Three classes of ground-state diketo tautomers, D1, D2 and D3 are investigated as alternatives to the A and Q forms discussed in the main text. Representative structures are shown in Figure S2. It is worth noting that only tautomer D1 is reasonable for molecules **1**-**4** whereas molecules **5** and **6** also possess D2 forms and molecules **7**-**8** also have an accessible D3 form where a pyrrole ring is formed. Fig. 6 shows the energies of the most stable D-type tautomer. This refers to D1 for **1**-**4**, to D2 for **5** and **6**, and to D3 for **7**-**8**. It is notable that the D3 tautomer, which forms a pyrrole ring, possesses enhanced stability meaning that these structures will most likely to be found as D3 tautomers.

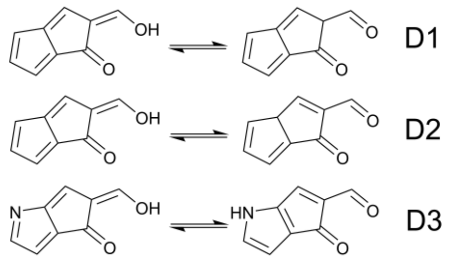

Figure S2. Chemical structures of 3 classes of tautomer investigated.

5. Further computational and experimental results

Table S28. Vertical energies (in eV) of o-hydroxybenzaldehyde (OHBA)[15], salicylic acid (SA)[16], 10-hydroxybenzo[h]quinoline (HBQ)[17], benzocyclobutenedione[18] and [2,2'-bipyridyl]- 3,3'-diol [BP(OH)$_2$][19] at the geometry of A(S_0) and Q(S_1) using the TDA/M06-2X/def2-TZVP and ADC(2)/def2-TZVPP methods as well as the experimental absorption maxima.

Mol. / Geo.	M06-2X	ADC(2)	Exp.
OHBA / A	4.07	3.99	3.9
OHBA / Q	3.32	2.49	2.5
SA / A	4.80	4.36	3.9
SA / Q	3.62	2.83	2.8
HBQ / A	4.06	3.66	3.26
HBQ / Q	2.58	1.68	1.98
Benzocyclobutenedione	5.08	4.71	4.48
[BP(OH)$_2$] / DE	4.31	3.92	3.65
[BP(OH)$_2$] / DK	3.18	2.54	2.43

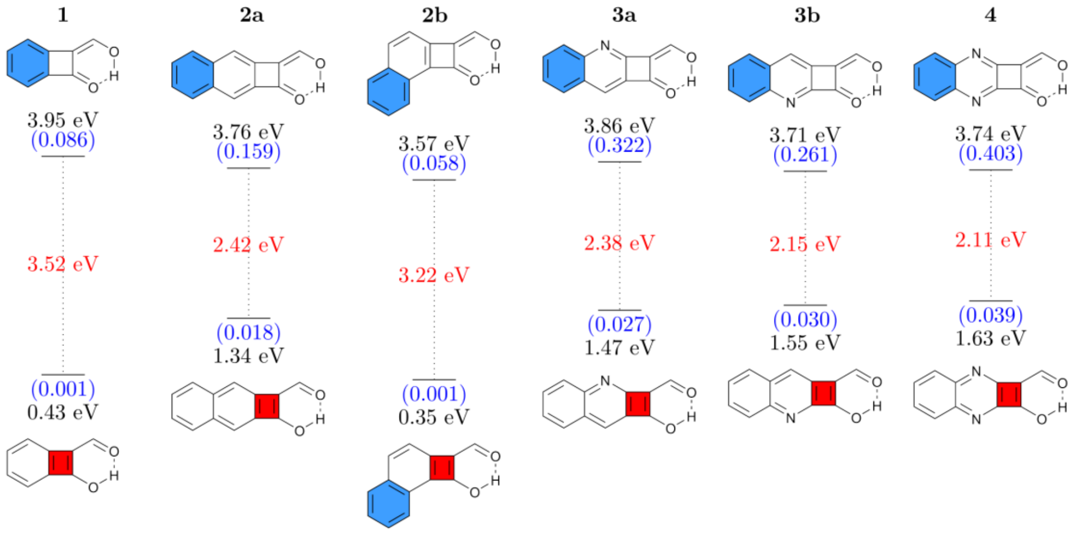

Figure S3. Vertical absorption energies in the Q form (top) and emission energies in the A form (bottom) at the M06-2X/def2-TZVP level for CBD

derivatives studied. Oscillator strengths (in parentheses, blue) and Stokes shifts (red) are given as well.

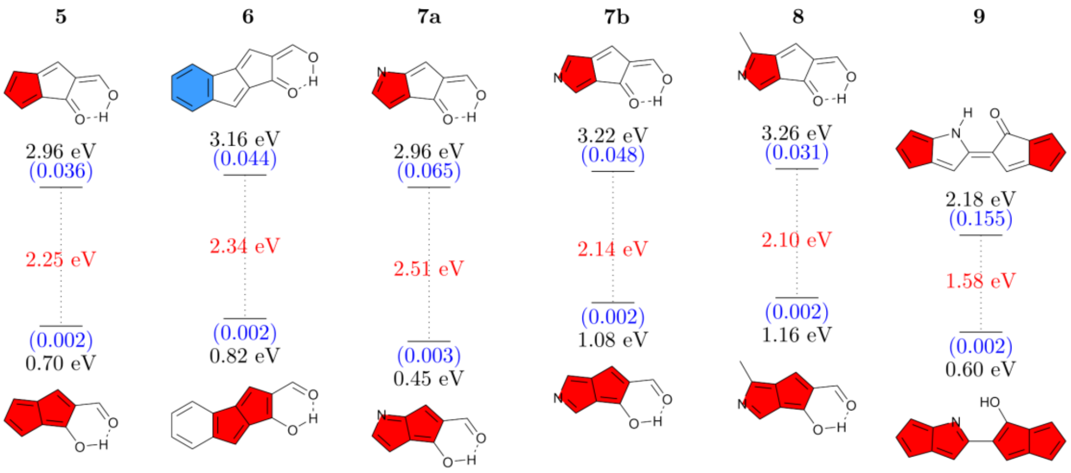

Figure S4. Vertical absorption energies in the Q form (top) and emission energies in the A form (bottom) at the M06-2X/def2-TZVP level for pentalene derivatives studied. Oscillator strengths (in parentheses, blue) and Stokes shifts (red) are given as well.

6. References

(1) P. V. R. Schleyer, C. Maerker, A. Dransfeld, H. Jiao, N. J. R. Van and E. Hommes, *J. Am. Chem. Soc.*, 1996, **118**, 6317.

(2) C. Adamo and V. Barone, *J. Chem. Phys*., 1999, **110**, 6158–6170.

(3) F. Weigend and R. Ahlrichs, *Phys. Chem. Chem. Phys.*, 2005, **7**, 3297–330.

(4) M. J. Frisch, G. W. Trucks, H. B. Schlegel, G. E. Scuseria, M. A. Robb, J. R. Cheeseman, G. Scalmani, V. Barone, B. Mennucci, G. A. Petersson, H. Nakatsuji, M. Caricato, X. Li, H. P. Hratchian, A. F. Izmaylov, J. Bloino, G. Zheng, J. L. Sonnenberg, M. Hada, M. Ehara, K. Toyota, R. Fukuda, J. Hasegawa, M. Ishida, T. Nakajima, Y. Honda, O. Kitao, H. Nakai, T. Vreven, J. J. A. Montgomery, J. E. Peralta, F. Ogliaro, M. Bearpark, J. J. Heyd, E. Brothers, K. N. Kudin, V. N. Staroverov, T. Keith, R. Kobayashi, J. Normand, K. Raghavachari, A. Rendell, J. C. Burant, S. S. Iyengar, J. Tomasi, M. Cossi, N. Rega, J. M. Millam, M. Klene, J. E. Knox, J. B. Cross, V. Bakken, C. Adamo, J. Jaramillo, R. Gomperts, R. E. Stratmann, O. Yazyev, A. J. Austin, R. Cammi, C. Pomelli, J. W. Ochterski, R. L. Martin, K. Morokuma, V. G. Zakrzewski, G. A. Voth, P. Salvador, J. J. Dannenberg, S. Dapprich, A. D. Daniels, O. Farkas, J. B. Foresman, J. V. Ortiz, J. Cioslowski and D. J. Fox, *Gaussian 09, Revision E.01*, Wallingford, CT, 2013.

(5) F. Plasser and F. Glocklhofer, *Eur. J. Org. Chem.*, 2021, **2021**, 2529–2539.

(6) F. Plasser, *J. Chem. Phys.*, 2020, **152**, 084108.

(7) W. Humphrey, A. Dalke and K. Schulten, *J. Mol. Graphics*, 1996, **14**, 33–38.

(8) E. Matito, M. Duran and M. Sol, *J. Chem. Phys.*, 2005, **122**, 014109.

(9) M. Giambiagi, M. S. D. Giambiagi, C. D. D. S. Silva and A. P. D. Figueiredo, *Phys. Chem. Chem. Phys.*, 2000, **2**, 3381–3392.

(10) P. Bultinck, R. Ponec and S. V. Damme, *J. Phys. Org. Chem.*, 2005, **18**, 706–718.

(11) T. A. Keith, *AIMAll (Version 19.10.12)*, 2019, TK Gristmill Software, Overland Park KS, USA.

(12) E. Matito, *ESI-3D: Electron Sharing Indexes Program for 3D Molecular Space Partitioning*, 2014, Institute of Computational Chemistry and Catalysis, Girona, Catalonia, Spain, 2014. http://iqc.udg.es/eduard/ESI.

(13) R. L. Martin, *J. Chem. Phys.*, 2003, **118**, 4775–477.

(14) Y. Zhao and D. G. Truhlar, *Theor. Chem. Acc.*, 2008, **120**, 215–241.

(15) S. ichi Nagaoka, N. Hirota, M. Sumitani, K. Yoshihara, E. Lipczynska-Kochany and H. Iwamura, *J. Am. Chem. Soc*, 1984, **106**, 6913–6916.

(16) D. D. Pant, H. C. Joshi, P. B. Bisht and H. B. Tripathi, *Chem. Phys.*, 1994, **185**, 137–144.

(17) C. Schriever, M. Barbatti, K. Stock, A. J. Aquino, D. Tunega, S. Lochbrunner, E. Riedle, R. de Vivie-Riedle and H. Lischka, *Chem. Phys.*, 2008, **347**, 446–461.

(18) K. Lohmann, Z. S. E. Biochem, G. Kerr, E. W. Huber and T. W. Sutherland, *J. Biol. Chem*, 1957, **233**, 3608.

(19) E. S. S. Iyer and A. Datta, *J. Phys. Chem. B*, 2012, **116**, 5302–5307.
